# Exploring the Learning Experiences of Non‐Medical Prescribing Among Podiatry Students on Clinical Placement

**DOI:** 10.1002/jfa2.70140

**Published:** 2026-02-25

**Authors:** Stephanie Haines, Hamna M. Khan, Sophia T. Pano, Simon Otter, Kim Holmes, Kate Carter

**Affiliations:** ^1^ Department of Podiatric Medicine and Surgery, School of Health and Clinical Sciences The University of Western Australia Perth Australia; ^2^ Health Sciences University, Parkwood Campus Bournemouth UK

**Keywords:** clinical placement, endorsement for scheduled medicines, non‐medical prescribing, podiatry students

## Abstract

**Background:**

Endorsement for scheduled medicines has been available to Australian podiatrists for over a decade. However, uptake and prescribing rates remain low. A key barrier is the limited number of endorsed prescribers, which restricts access to mentorship opportunities for podiatrists seeking endorsement. While existing literature explores practicing podiatrists' perspectives on endorsement, the views of students regarding prescribing remain underrepresented. This study aimed to explore final‐year podiatry students' experiences with non‐medical prescribing during clinical placements.

**Methods:**

This qualitative study purposively recruited final‐year podiatry students from the University of Western Australia. Semi‐structured interviews were undertaken to explore participants' experiences of non‐medical prescribing during clinical placements, which were audio‐recorded, transcribed verbatim and analysed using constant comparative analysis until data saturation was reached. Data recorded prior to interview included demographic information, academic characteristics were determined using self‐reported questionnaires to describe motivation, self‐efficacy and mental wellbeing and clinical logbook records were retrospectively reviewed for data on prescribing encounters recorded by students during clinical placements.

**Results:**

Fifteen participants aged between 23 and 46 years were recruited. Retrospective clinical logs showed that 631 of 7613 cases (8.3%) involved prescription of a scheduled medicine, with most prescribing opportunities occurring in public health settings. Six global themes relating to prescribing experiences emerged: limited exposure, confidence, learning experiences, proposed improvements, pursuit of endorsement and future career impacts. Relationships between themes were developed based on key concepts, particularly scarcity of endorsed prescribers, and limited amount and variety of prescribing encounters.

**Conclusion:**

This study demonstrated that although podiatry students experienced limited exposure to endorsed prescribing and low self‐confidence in prescribing ability, they continued to see value in non‐medical prescribing with a willingness to pursue endorsement in the future. Future implications to address limited student exposure to prescribing due to shortage of endorsed podiatrists may facilitate the uptake of endorsement for scheduled medicines.

**Abbreviations**
CoP: Communities of practice
EPs: Endorsed podiatrists
ESM: Endorsement for scheduled medicines
NMP: Non‐medical prescribing
WIL: Work integrated learning

## 1 Introduction

The health system reform in Australia that introduced non‐medical prescribing (NMP) by allied health professionals (AHPs), including podiatrists, was intended to provide more timely, cost‐effective and convenient access to medicines for patients, which would in turn improve health outcomes [1, 2, 3, 4]. Podiatrists and other AHPs wishing to acquire NMP prescribing rights must first obtain endorsement for scheduled medicines (ESM). ESM involves registration by the relevant health practitioner board that will recognise a practitioner as qualified to prescribe certain restricted (i.e., scheduled) medicines. Once obtained, this authority allows endorsed practitioners to prescribe a broad range of medicines. Other health professions, such as nurse practitioners, pharmacists and optometrists, have demonstrated high levels of uptake of the specialised training required to prescribe specific scheduled medicines [1, 2, 3, 4]. Despite podiatry having the option to take up ESM through the National Registration and Accreditation Scheme for over a decade, uptake of prescribing rights remains low within the profession across both public and private sectors, with only 4.88% (*n* = 303) of Australian podiatrists currently qualified for NMP via ESM [5].

ESM for podiatrists can be attained either as part of a tertiary qualification (e.g., degree or postgraduate certificate) with accredited pharmacotherapeutic training (known as Pathway A) or a post‐registration case portfolio documenting 100 h of supervised prescribing practice and at least 40 prescribing cases (known as Pathway B) [6, 7, 8, 9, 10]. Currently, there is one Australian podiatry degree program that offers Pathway A and two that offer Pathway B through part‐time post‐registration courses. Therefore, the majority of approximately 300 podiatrists currently graduating annually Australia‐wide are not graduating with NMP rights [11], if they wish to obtain NMP rights they can obtain ESM through Pathway B.

Published Australia‐based national studies have identified key barriers to obtaining ESM among registered podiatrists via Pathway B, which include the cost of training, difficulty achieving the portfolio requirements, limited access to endorsed clinical supervisors and inadequate structured clinical training [10, 12, 13]. Despite major challenges recognised in previous studies among practicing podiatrists, there has been limited focus on the predisposition of students to seek future ESM and its potential impact on workforce planning to build capacity for podiatry endorsement.

Published recommendations to ameliorate the challenges to obtaining endorsement have suggested workforce strategies that foster prescribing‐focused communities of practice (CoP) to improve professional support and build capacity for podiatry endorsement [10]. Similarly, in the context of pre‐registration education, work‐integrated learning (WIL)—learning obtained on placement in a workplace—provides students with structured clinical placements to gain experience of the prescribing process by observing endorsed podiatrists (EPs) and medical prescribers. Evidence shows that exposure to CoP during WIL by students strengthens professional identity, improves clinical confidence and fosters motivation to pursue extended scope qualifications such as prescribing rights [14, 15]. Australian podiatry degree programmes nationwide have embedded NMP components alongside WIL principles to maximise prescribing‐related opportunities in clinical settings for students, with the aim of increasing engagement with ESM in the future podiatry workforce and building capacity and capability within the programme to meet the accreditation standards for endorsement of registration for scheduled medicine in the future (Pathway A) [16]. It is envisaged that ESM will be part of all Australian podiatry degree programmes in the next 5 years.

While previous studies have examined barriers and facilitators to ESM among registered podiatrists [7, 10, 12], there has been limited investigation of students' learning experiences and their impact on perceptions of podiatric prescribing, whether positive, negative or neutral. Exploring the prescribing‐related experiences of soon‐to‐graduate podiatry students during WIL would provide insight into their attitudes towards NMP and future ESM. These insights could inform strategies to improve prescribing‐related learning and support increased rates of training for ESM in the emerging workforce. This is relevant to universities aiming to integrate ESM requirements within accredited podiatry degree programs through observation and supervised prescribing practices, thereby promoting ESM and professional advancement.

The aim of this study was to explore the WIL learning experiences among final‐year podiatry students of prescribing‐related encounters during clinical placements and, in doing so, examine student predisposition to seek, or not, future ESM and to inform potential curriculum strategies to better support the future workforce.

## 2 Methods

### 2.1 Study Design

A qualitative research approach was used to capture in‐depth accounts of final‐year podiatry students' learning experiences of NMP during clinical placements. Semi‐structured one‐to‐one interviews were used to gain an understanding of their individual learning experiences and to encourage disclosure of personal opinions [17]. An interview guide (Supporting Information S1) was developed based on a review of relevant literature [10, 12, 18, 19] and refined by researchers experienced in qualitative methods and NMP research [KC and SO]. Optional probes were included to facilitate discussion and maintain focus on prescribing processes [20]. The guide was piloted with two podiatry students from a different cohort outside of the main study sample, with wording adjusted to improve clarity and construct validity.

### 2.2 Participants

Purposive sampling was used to recruit final‐year podiatry students enrolled at the University of Western Australia (UWA). Recruitment commenced in October 2024 (in the final semester of the student's final‐year) with all pre‐registration students having completed the WIL component of the podiatry degree programme. A potential total sample size of 16 eligible participants was considered sufficient given recommendations that suggest qualitative data saturation can be achieved with 6–12 participants [21].

Recruitment materials were distributed through the UWA administrative office as an independent third‐party to minimise any undue influence on potential participants' decision to take part in the study. Although potential participants were enrolled in a different cohort from the student researchers, it was made clear in the study information and by investigators that participation was completely voluntary and their decision to participate or withdraw would not affect their relationships with the program or the university. All participants provided written informed consent prior to data collection.

### 2.3 Data Collection

Prior to interview, demographic information was collected including age, gender, ethnicity and work status. Academic characteristics, including academic motivation, self‐efficacy and physical and mental wellbeing, were assessed using the Short Academic Motivation Scale (SAMS), the General Self‐Efficacy Scale (GSE) and the Salutogenic Health Indicator Scale (SHIS), respectively. Each of which are self‐reported questionnaires validated for use in healthcare student populations [22, 23, 24], with lower scores indicating worse status. Academic characteristics, including motivation, self‐efficacy and well‐being, were collected to describe the psychological disposition of participants, which are intrinsic factors that influence and possibly determine the general outlook and morale of the sample.

Clinical logbook records were retrospectively reviewed (SO, HK and SH) for data to objectively describe the frequency, work sector, geographical location and type of prescribing encounters recorded by students during clinical placements. Consent was sought from all participants to review their clinical logbook in Meditrek, an online data platform used by students to record a minimum of 4 patient encounters per day during clinical placement to fulfil academic program requirements. Clinical logbook records over a 10‐month period (from January to October 2024) of participants' final year were collated and fully anonymised by an independent UWA administrator prior to analysis.

Semi‐structured interviews were conducted face‐to‐face in a private room to explore final‐year podiatry students' perspectives on NMP. The interview guide used open‐ended questions to encourage detailed descriptions. All interviews were conducted by the same researcher (HK) and supported by a second researcher (SP), both trained in qualitative methods and interview techniques. Interviews lasted approximately 30 min, were audio‐recorded, transcribed verbatim and de‐identified. Data were collected between October and November 2024.

### 2.4 Data Analysis

Demographic, academic characteristics and clinical logbook data were summarised using descriptive statistics. Qualitative data were analysed using a constant comparative method [25], which combines inductive category coding with a simultaneous comparison of all meaningful units. Researchers (HK and SP) read the transcripts independently multiple times to aid triangulation. Meaningful units within the data, including phrases and concepts, were assigned codes that aligned with the study's focus. Codes were iteratively refined, compared and then grouped by shared meanings to form themes, presented as theme maps and rich textual description with verbatim quotes. This methods approach required the researchers to remain close to the data in qualitative description [26]. Data saturation, defined as no new codes, was determined during data collection and from data analysis [21]. Emergent themes were reviewed by the research team and subsequently verified by 4 participants with no changes recommended.

## 3 Results

### 3.1 Demographic and Academic Characteristics

Demographic and academic characteristics are summarised in Table 1. Of a total sample size of 16 eligible students, 15 final‐year podiatry students were recruited, with a mean (SD) age of 27 (7.3) years. The majority of participants were female (60%), lived in their family home (67%), had never been married (73%) or had children (80%) and could speak a language other than English (67%).

**TABLE 1:** Demographic and academic characteristics of final‐year podiatry student participants.

Participant characteristics	Total participants (*n* = 15)
Female (*n*, %)	9 (60)
Male (*n*, %)	6 (40)
Age in years (mean, SD)	27 (7.3)
Ethnicity (*n*, %)	
Australian European	6 (40)
Southern and Southeast Asian	3 (20)
Chinese	2 (13)
Other	2 (13)
European	1 (7)
Middle Eastern/North African	1 (7)
Language other than English spoken at home (*n*, %)	
Yes	10 (67)
No	5 (33)
Marital status (*n*, %)	
Never married	11 (73)
Married or de factor relationship	4 (27)
Children (*n*, %)	
No	12 (80)
Yes	3 (20)
Work status (*n*, %)	
Unemployed	7 (48)
Casual paid work	5 (33)
Part‐time paid work	3 (20)
Living arrangement (*n*, %)	
Lives with parents in family home	10 (67)
Lives with partner	4 (27)
Lives with housemates	1 (7)
Academic characteristics (mean, SD)	
Short academic motivation scale (scores range 0–6)	3.6 (1.0)
General self‐efficacy scale (scores range 0–40)	31.1 (0.8)
Salutogenic health indicator scale (scores range 12–72)	37.9 (2.9)
Grade point average (GPA) (*n*, %)	
> 6.0 (good)	8 (53)
5.0–6.0 (moderate)	6 (40)
< 5.0 (poor)	1 (7)
Future workplace intention (*n*, %)	
Private practice	14 (93)
Public sector	6 (40)
Regional or remote	2 (13)
International	1 (7)
Hours per week on clinical placement (mean, SD)	36 (5.7)
Placement level of involvement (*n*, %)	
Mainly manages cases	7 (47)
Participates more than observes cases	7 (47)
Mainly observes cases	1 (7)

Abbreviation: SD, standard deviation.

Self‐reported academic characteristics indicated moderate levels of academic motivation and general well‐being, and high self‐efficacy among participants and over half of respondents (53%) had a high grade point average (GPA 6.0 or greater). Most participants (93%) expressed an intention to work in the private sector after graduation. Participants reported an average total duration of 36 hours per week on clinical placement, with most students either fully or partially involved in the management of patient cases, inclusive of prescribing and non‐prescribing encounters.

### 3.2 Clinical Logbook Data

**FIGURE 1:**
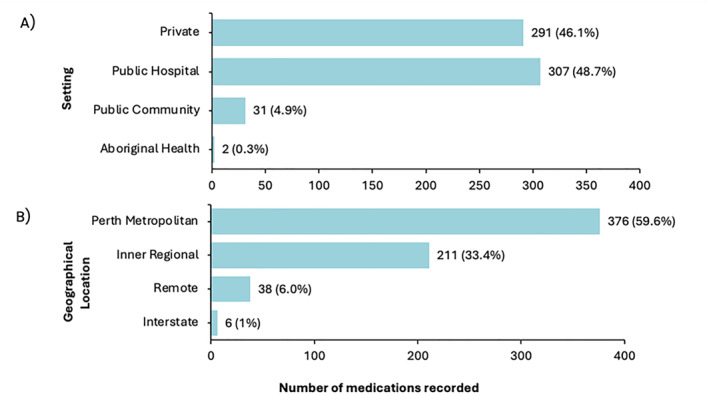
Frequency of medications recorded in clinical logbooks by final‐year podiatry students during clinical placement between January and October 2024, stratified by (A) setting and (B) geographical location. *Geographical location was defined using the Australian Statistical Geography Standard (ASGS), which defines relative remoteness using the Accessibility and Remoteness Index of Australia [27].

A total of 631 (8.3%) recorded clinical logbook encounters involved prescribing, with 49% taking place in public hospitals (Figure 1A), and most occurring within the Perth metropolitan area (Figure 1B).

**FIGURE 2:**
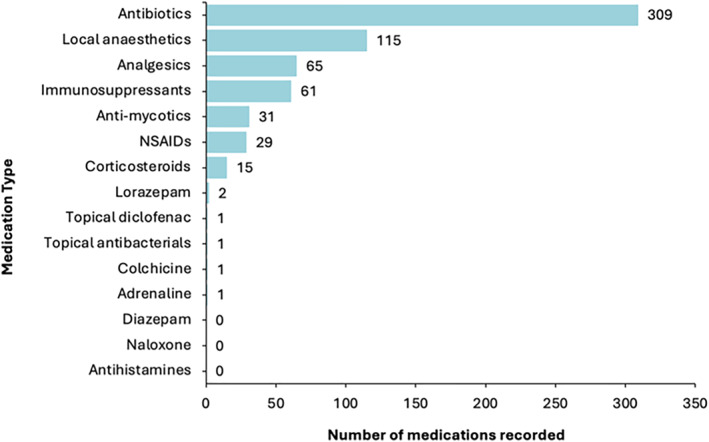
Frequency of medication types recorded in clinical logbooks by final‐year podiatry students during clinical placements between January and October 2024. (NSAIDS *non‐steroidal anti‐inflammatories*).

Antibiotics comprised the majority (309, 49%) of all logged medications, followed by local anaesthetics (115, 18%), analgesics (65, 10%) and immunosuppressants (61, 10%) (Figure 2).

## 4 Qualitative Data

### 4.1 Themes

**FIGURE 3:**
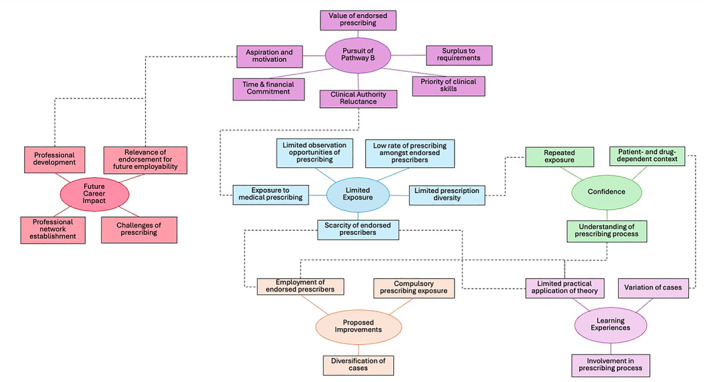
Thematic map featuring six global themes and respective subthemes, and their interconnected relationships that were verified by participants.

Six global themes emerged and are listed with their subthemes in Table 2. Relationships between subthemes are depicted in Figure 3. Exemplars from the transcripts were identified to support each of the themes.

**TABLE 2:** Global themes and respective subthemes on the learning experiences of NMP among final‐year podiatry students during clinical placement.

Global themes	Subthemes
1. Limited exposure to endorsed prescribing	Limited observation opportunities
	Low prescribing rate
	Limited prescription diversity
	Exposure to medical prescribing
	Scarcity of endorsed prescribers
2. Confidence in prescribing abilities	Understanding of prescribing process
	Repeated exposure
	Context‐dependent confidence
	Limited integration of prescribing theory into practice
3. Learning experiences of prescribing	Variation of cases
	Involvement in prescribing process
4. Proposed improvements to placement process	Employment of endorsed prescribers
	Compulsory prescribing exposure
	Diversification of cases
5. Pursuit of endorsement	Value of endorsed prescribing
	Surplus to requirements
	Priority of clinical skills
	Aspiration and motivation
	Clinical authority reluctance
	Time and financial commitment
6. Future career impact	Professional development
	Relevance and future employability
	Network establishment
	Challenges of prescribing

#### 4.1.1 Theme 1: Limited Exposure to Endorsed Prescribing

The majority of participants perceived limited exposure to prescribing practices during clinical placements. This was mainly attributed to insufficient prescribing opportunities, which referred to clinical encounters where a medication could be prescribed, during which students either observed prescribing by endorsed prescribers or medical practitioners or had their own prescriptions reviewed and approved by an endorsed prescriber. When opportunities did occur, participants reported infrequent prescribing activity and low diversity of prescribing practice from a narrow range of medications and podiatric conditions.

… in terms of prescribing for Podiatry, I definitely feel like there hasn’t been enough podiatry‐specific exposure, for example fungal nails or other conditions like that.

P06

We don't really see many cases that actually need to prescribe.

P12

Very rare… throughout these 3 years, I've just basically witnessed like 2 prescriptions and that's about it.

P14

Participants linked the limited availability of prescribing opportunities to the low number of EPs in the workforce. Not all EPs offer clinical placements and those who do often work in practices alongside non‐endorsed practitioners, which reportedly contributed to limiting exposure of students to prescribing practice.

… not many places we go to, there’s going to be an endorsed podiatrist.

P05

Prescribing exposure most frequently observed was doctor‐led within hospital settings, which participants perceived as having low relevance to podiatric NMP.

I feel like there's a moderate amount of observation in hospitals, but it's not really from the perspective of a podiatrist.

P07

No, and it wasn't relevant because it was prescribing things not on our schedule.

P16

Participants identified time constraints and limited opportunities to practise prescribing rationale and processes as key barriers to completing curriculum‐mandated practical assessments during clinical placements.

…we’ve got an ESM capability and no one's able to get it signed off because there’s no one [available] that can sign it off.

P07

[Few] supervisors in clinic are endorsed, and then when on placement they don't have the time.

P15

In the public health setting, participants perceived there to be an underutilisation of prescribing rights by EPs by transferring prescribing responsibilities to medical prescribers. This was interpreted as either a professional hierarchy of medical practitioners that limited NMP practices or EPs working as part of a multidisciplinary team with shared decision‐making.

I think I was surprised in hospital about how hesitant [podiatrists] are to prescribe.

P07

#### 4.1.2 Theme 2: Confidence in Prescribing Abilities

Participants' confidence in their ability to prescribe varied, with most reporting lower confidence levels due to limited experience and understanding of NMP practices. Those with moderate‐to‐high confidence noted that it was confined to frequently encountered medications (such as antibiotics) or situations involving supervision rather than independent prescribing.

At the moment, with the amount of skills and exposure that I have, I don't think I would be entirely confident in prescribing on my own.

P06

I feel more confident if I were to prescribe antibiotics. Because it's done so often.

P08

Participants recognised challenges in translating theoretical knowledge into practice, which negative impacted on students' confidence and learning experience.

#### 4.1.3 Theme 3: Learning Experiences of Prescribing

Participants' NMP learning experiences varied in type and complexity across clinical settings and healthcare sectors. Hospital placements provided a broader range of prescribing experiences, including medical prescribing, which was valued for demonstrating clinically safe practice guided by recognised resources such as the Therapeutic Guidelines (eTG), Monthly Index of Medical Specialities (MIMS) and the Australian Medicines Handbook (AMH).

Watching the doctors do it is great. It's actually good to see how they work within the guidelines.

P01

It was hammered into us to know where to find the answers … different resources like MIMS and the AMH. That very much matches what I saw in practice… they will go on eTG, [and] MIMS and they're making sure they've got the right indicated drug.

P15

For some participants, observing safe and effective NMP reinforced the value of podiatric endorsement.

With the clinical placements, they've definitely highlighted the importance of getting prescribing rights and also the benefit of having a lot more podiatrists having prescribing rights.

P02

While most participants reported valuable learning during clinical placements, those dissatisfied with the limited number of prescribing opportunities voiced perceived learning gaps. These included key components of the prescribing process, clinical rationale behind drug selections and unique prescribing considerations of comorbidities and drug interactions.

There was no detail around why they choose a particular antibiotic over another one, particularly when it varied from the eTG or what the decision‐making processes were behind their choices.

P10

A commonly reported experience was the limited involvement of participants by endorsed practitioners in the prescribing process, which resulted in students feeling underequipped to prescribe, even in similar potential cases in the future.

I haven’t really done anything myself. I’m just in the room, watching it take place.

P04

I haven’t prescribed or written a prescription for a patient and then had someone that was endorsed actually go through it. The only experience I’ve had is setting out prescriptions in assignments, I haven’t done it for a real patient.

P13

#### 4.1.4 Theme 4: Proposed Improvements to Placement Process

All participants identified areas for improvement in prescribing‐related learning during clinical placements. Most recommended greater EP involvement in academic settings and utilisation of complex, realistic patient cases to facilitate exposure and proactive involvement of students in prescribing processes.

In an ideal world, I would prefer to see us being paired with EPs when we're sent off to our private rotations. It's difficult to try and accommodate that given the low numbers though.

P03

I think they just need to … get more supervisors that are endorsed and that way when we do have a patient that needs something we're not like ‘Oh go see your GP’.

P13

Of the 15 participants, 14 suggested broadening clinical case diversity and podiatry‐specific prescribing, noting antibiotics were the most frequently observed medication and reflecting the mainstay of routine podiatric practise.

Giving more access to more cases outside of antibiotics, even pain relief, analgesics, would be really good. Other than the boring stuff.

P04

…having the opportunity to observe ESM meds in podiatric settings would make a significant difference.

P10

#### 4.1.5 Theme 5: Pursuit of Endorsement

Positive placement experiences and meaningful interactions with EPs revealed participants' interest in pursuing ESM after graduation.

Definitely a pathway I’m interested in going down, especially with now not being able to graduate with endorsement. It's a good way to see prescribing and also potentially make a connection with the prescriber so they could possibly be a mentor.

P02

The types of patients… and level of treatments … It was inspiring being able to see [podiatrists' capabilities]. It made me want to pursue endorsement.

P03

Meanwhile, other participants identified several barriers to pursuing endorsement, such as financial constraints, intensity of the process and reluctance to assume the additional responsibilities it entails.

There’s a lot more training, it's basically a part‐time job to factor in.

P11

We really need to have a high standard we need to thoroughly prove that we are safe and capable prescribers, because we cannot have any mistakes.

P15

Besides the fact that when you do it, it's private as well, so it's more costly to the patient.

P16

While a few participants considered NMP a desirable but non‐essential credential, the vast majority perceived ESM as a valuable credential that extends podiatric scope, supports professional autonomy and promotes patient continuity and access to care.

It's a treatment modality. It’s more scope of practice.

P01

It saves the patients the trip of having to go to a medical practitioner.

P02

There aren’t many doctors in [remote] regions, so the podiatrist often ends up being the patient’s only point of contact.

P03

Wouldn't it be great if we could just prescribe. Even to save the patient the trip, the cost, the logistics, the anxiety of trying to get in to see a GP.

P15

[From] what I've observed and also discussed with general podiatrists, if they were able to prescribe, the majority of them said it'd be nice, but they definitely can still survive and they go through like the GPs to prescribe.

P02

Prioritising the development of clinical skills, some participants indicated that they did not plan to pursue endorsement immediately after graduation, choosing instead to focus on building practical competence and learning their role. They also noted that the value and relevance of endorsement may depend on their future job setting, with it perceived as most beneficial in hospital environments.

Probably unlikely for the next 2 years, maybe a year just to get my normal skills up first.

P11

#### 4.1.6 Theme 6: Future Career Impact

All participants recognised endorsement as a facilitator of career advancement, perceiving it to improve professional competitiveness and long‐term employability within the podiatric workforce.

I feel like it's a niche in podiatry. It's a good way to differentiate yourself from other general podiatrists.

P02

I think podiatrists would be left behind … fast forward 20 years and probably most young podiatrists have endorsement.

P15

Participants felt that a wider professional network of EPs would broaden access to future mentorship, foster meaningful professional relationships and support ongoing professional development.

Throughout placement experiences, and we're also linked up with a network of podiatrists who know someone who's endorsed, or you know, can link me up with people who are able to mentor.

P03

I think it's very important for podiatrists to become endorsed. I think it helps with your management of cases, as well as adherence to the treatment plans that you provide.

P08

Barriers to attaining endorsement were reported to be the substantial time commitment and training requirements, and challenges associated with prescribing itself stemmed from the added responsibility for patient safety felt by participants.

There’s a lot more responsibility that comes with prescribing.

P16

## 5 Discussion

Despite recognised challenges with the limited availability of EPs to support NMP development, most final‐year podiatry students remained highly motivated to pursue ESM post‐registration, valuing its potential to improve patient care and advance the profession. This is the first Australian‐based study to examine NMP from the perspective of pre‐registration students rather than practising podiatrists. This student‐centred lens offers new insight into how endorsement is perceived during pre‐registration training and highlights potential educational and health system strategies that could better support the future uptake of prescribing rights in podiatry.

Low rates of prescribing by EPs and low diversity of prescribing practice (i.e., a narrow range of medications) and podiatric conditions were reported by final‐year students during clinical training. This study finding was supported by the clinical logbook data showing antibiotics and local anaesthetics for diabetic foot ulcers and ingrown toenails, respectively, to be the most frequently prescribed (67%). Similarly restricted patterns in NMP among practicing podiatrists nationally were published in 2022 [12], which may suggest that prescribing practices and student opportunities have seen little change in the years since. Published studies in podiatry and other allied health professions and nursing have shown that cautious or infrequent prescribing has been linked to insufficient practical experience, preventing skill consolidation and successful implementation of prescribing rights [12, 19, 28, 29, 30, 31, 32, 33]. Current study findings may reflect this broader cycle of persistently low prescribing rates among EPs leading to limited student exposure to prescribing and reduced mentorship opportunities, causing low student confidence in future prescribing ability, which often results in a reluctance to prescribe or risk eversion to prescribing. Collaborative efforts between universities, professional bodies, healthcare organisations and employers are required to coordinate clinical training pathways to support EPs to implement and maintain prescribing competencies in practice.

Over a 10‐month period, prescribing encounters accounted for 8.3% (*n* = 631) of clinical logbook entries. Although participants in the current study regarded this level of exposure to prescribing practice as insufficient, currently there are no professional recommendations on the frequency of prescribing encounters required for pre‐registration podiatry students. Indeed, a new Australia‐based study to be conducted by Queensland University of Technology funded by the Australasian Council of Podiatry Deans through the Australian Podiatry Education and Research Foundation is seeking to establish national consensus on podiatry‐specific capabilities for prescribing scheduled medicines [34] unpublished. This highlights the lack of existing guidance on the specific knowledge and skills required to demonstrate competent prescribing practice and on the teaching and assessment of podiatry prescribing skills at university. Five of seven Australian university podiatry programmes are collaborating on this unpublished research, which suggests wide recognition among course providers that a better understanding of the needs of podiatrists to prescribe medicines is required to inform curriculum design for university courses that are, or will be, training podiatrists to prescribe medicines.

In the public hospital setting, students reported their most positive learning experiences of prescribing and clinical logbook data showed the highest rates of prescribing. In contrast to the UK where podiatry services are well established in the public health system, enabling podiatrists to routinely prescribe medicines in specialised roles within diabetes, musculoskeletal and rheumatology services [1, 4, 35], only 35% of Australian podiatrists work in the public sector. Despite substantial unmet footcare needs [36, 37], a recent Australian‐based national study found that zero podiatrists were employed in public rheumatology departments [37]. Furthermore, the density of the Australian podiatry workforce is more concentrated in metropolitan and most advantaged areas across each state and territory [38], with approximately only 6% of the Australian podiatry workforce located in outer regional or remote settings [39]. Inadequate access to the podiatry workforce and its services is thought to contribute to health disparities [40], limiting workforce prescribing capacity to address the footcare needs of the population. Government initiatives are required to support the minimum staffing requirements of podiatry services in the Australian public health system and to recognise the value of embedding NMP within public healthcare service provision.

Repeated opportunities to observe medical prescribing of antibiotics in the hospital settings were reported by students to be particularly valuable for learning and increasing confidence in their prescribing knowledge of antibiotics. This confidence may be attributed to repeated situational exposure and observational learning, both recognised as effective for consolidating prescribing knowledge [30, 41]. In the United Kingdom, structured opportunities for interprofessional observation of prescribing practices are required in the UK NHS NMP training [1]. Embedding interprofessional education models to provide medical and non‐medical prescribing learning opportunities would increase exposure and access to mentorship, especially given the shortage of EPs. In Western Australia, 8% of podiatrists are endorsed [5] and approximately one‐third (16, 33%) of these provide student supervision in the academic clinical setting or during clinical placement. Healthcare organisations could facilitate a mentorship scheme by creating a register of qualified medical and non‐medical prescribers who would be willing to undertake a supervisory role for students and newly qualified colleagues [42]. In addition, where students reported limited involvement in the prescribing process, provision of structured post‐placement debriefing or action learning sets may help to maximise learning opportunities and address challenges related to developing professional competencies in NMP and ESM through clinical education.

Participants remained highly motivated to pursue endorsement training despite limited prescribing exposure, low confidence in prescribing knowledge and anticipated training barriers. Motivation appeared driven by participants' perceptions of NMP to improve patient‐centred care, professional scope and employability. Similar motivations have been reported among both early‐career and established non‐medical prescribers, who often view NMP as means to improve patient outcomes [10, 13, 28]. However, such perceptions are not universal and vary according to professional background, experience and practice environment [10, 13, 28]. Systematic reviews in NMP have indicated that novice prescribers may experience anxiety, low confidence and underpreparedness, factors that may impede their ability to implement prescribing post‐qualification [13, 29]. In the United Kingdom, post‐registration NMP students found that additional mentorship was an effective mechanism to help implement prescribing successfully post‐qualification [42]. Collectively, these findings reveal a disconnection between strong student motivation and the limited supervisory capacity to support the transition of prescribing theory into practice. Early, structured and ongoing mentorship may help sustain motivation, build confidence and strengthen ESM training uptake among future podiatrists. Future longitudinal research to investigate the uptake of ESM training among the current study cohort would clarify whether enthusiasm for NMP persists, or if external factors, such as time constraints, mentorship access and previous WIL experiences, determine successful endorsement.

This study was a valuable initial exploration of students' experiences of NMP in practice and their motivations for future uptake of ESM in podiatry. This study provided an important description of frequency and patterns of NMP observed by students in practice, which reflected national prescribing patterns of EPs previously reported [12].

Limitations of this study include the small sample size drawn from a single institution. Although data saturation was reached and study findings were confirmed through participant verification, the small sample limits generalisability and warrants replication across other podiatry programs. Prescribing encounters may be underreported given that clinical logbook recordings were not mandatory for all patients encounters during clinical placements, with a minimum of four entries required per day. In addition, students may not have recorded cases where the decision was made not to prescribe as a prescribing encounter, not considering it a learning opportunity of the prescribing cycle. For example, while clinical logbook data showed the highest rates of prescribing in the public health setting, students perceived there to be an underutilisation of prescribing rights by EPs, which may have reflected multidisciplinary team decision‐making on whether or not to prescribe a medication. However, since the current study, academic course requirements have changed to include the recording of all clinical encounters during clinical placement, and thus future work using the clinical logbook records would provide a more comprehensive description of prescribing practices observed.

## 6 Conclusion

This study provides new insight into how final‐year podiatry students experience and engage with NMP during clinical placements; a perspective largely underrepresented in existing literature. Despite limited prescribing exposure in practice, students consistently recognised the value of NMP and expressed strong motivation to pursue endorsement training post‐registration to improve patient care and professional scope. Study findings highlight systemic barriers within Australian healthcare, which narrows prescribing capacity and potentially the future growth of podiatric NMP.

## Author Contributions

**Stephanie Haines:** data curation, investigation, formal analysis, writing – original draft, writing – review and editing. **Hamna M. Khan:** data curation, investigation, formal analysis, visualisation, writing – original draft, writing – review and editing. **Sophia T. Pano:** data curation, investigation, formal analysis, visualisation, writing – original draft, writing – review and editing. **Simon Otter:** methodology, validation, writing – review and editing. **Kim Holmes:** conceptualisation, methodology, project administration, supervision, writing – review and editing. **Kate Carter:** conceptualisation, methodology, project administration, supervision, writing – review and editing.

## Funding

The authors have nothing to report.

## Ethics Statement

This study was approved by the University of Western Australia Human Research and Ethics Committee (2024/ET000437).

## Consent

All participants provided informed consent prior to data collection.

## Conflicts of Interest

The authors declare no conflicts of interest.

## Supporting information


Supporting Information S1

## Data Availability

The data that support the findings of this study are available from the corresponding author upon reasonable request.
