# Supplementary material for: Exploring the Learning Experiences of Non‐Medical Prescribing Among Podiatry Students on Clinical Placement
Source: J Foot Ankle Res. 2026 Feb 25;19(1):e70140. doi: 10.1002/jfa2.70140 (PMC12935556; doi:10.1002/jfa2.70140)
Supplement: Supplementary file 1 — Supporting Information S1 [file JFA2-19-e70140-s001.docx]

**Supplementary Information**

**Interview guide**

1. **How often did you get to observe and learn about endorsed prescribing during your clinical placements?**

Probes:

How much experience did you get on placement?

Did you feel the amount of exposure was appropriate? If not, why not? If so, why?

1. **Did you request to be placed with an endorsed prescriber for your clinical placements?**

Probes:

If not, why not?

If so, why?

1. **What is your opinion about endorsed prescribing in your clinical training?**

Probes:

Can you explain why you feel this way?

1. **Can you tell me about your learning experiences of endorsed prescribing during your clinical placement?**

Probes:

What kinds of prescribing encounters did you experience, if any?

What kinds of learning opportunities did you encounter, if any?

Can you tell me about any positive experiences?

Can you tell me about any negative experiences?

Are there any factors that contributed to this?

1. **Can you describe how exposure to endorsed prescribing has impacted on your knowledge of the prescribing process?**

Probes:

What areas or steps of the prescribing process did you learn about?

How has it impacted on your knowledge of pharmacological management?

Can you explain in what ways?

Can you give an example?

1. **What were your experiences with an endorsed prescriber on clinical placement?**

Probes:

Why do you feel this is?

How have these experiences impacted on your learning?

1. **From your experience of endorsed prescribing, what opportunities have you identified to prescribe in your clinical practice?**

Probes:

Can you give an example?

How have these experiences impacted on your learning?

1. **How confident do you feel applying your learning of endorsed prescribing into your own practice in the treatment of podiatric conditions?**

Probes:

How likely are you to use Endorsed Prescribing in your own practice?

If not, why not? If so, why?

Can you explain why that is?

1. **How likely are you to pursue endorsement when you graduate based on your experiences on placement?**

Probes:

Are there any factors that contributed to this?

Can you explain why you feel this way?

1. **Has your perspective of endorsed prescribing changed since your experience on clinical placements?**

Probes:

In what ways?

Why do you feel this is?

1. **How well does your pharmacology curriculum match what you see in practice?**

Probes:

Can you give an example?

1. **In what ways could your learning experience of endorsed prescribing during clinical placement be improved?**

Probes:

Can you give an example?

What clinical learning activities or training opportunities would help you?

How could your level of knowledge and understanding have been improved?

1. **Is there anything else that we have not already talked about that you would like to add concerning the experiences you have had with endorsed prescribing related to your clinical placement?**
